# A Systematic Literature Review of Studies Analyzing Inequalities in Health Expectancy among the Older Population

**DOI:** 10.1371/journal.pone.0130747

**Published:** 2015-06-26

**Authors:** Benedetta Pongiglione, Bianca L. De Stavola, George B. Ploubidis

**Affiliations:** 1 Medical Statistics Department, Faculty of Epidemiology and Population Health, London School of Hygiene and Tropical Medicine, University of London, London, United Kingdom; 2 Centre for Longitudinal Studies, Institute of Education, London, United Kingdom; Karolinska Institutet, ITALY

## Abstract

**Aim:**

To collect, organize and appraise evidence of socioeconomic and demographic inequalities in health and mortality among the older population using a summary measure of population health: Health Expectancy.

**Methods:**

A systematic literature review was conducted. Literature published in English before November 2014 was searched via two possible sources: three electronic databases (Web of Science, Medline and Embase), and references in selected articles. The search was developed combining terms referring to outcome, exposure and participants, consisting in health expectancy, socioeconomic and demographic groups, and older population, respectively.

**Results:**

Of 256 references identified, 90 met the inclusion criteria. Six references were added after searching reference lists of included articles. Thirty-three studies were focused only on gender-based inequalities; the remaining sixty-three considered gender along with other exposures. Findings were organized according to two leading perspectives: the type of inequalities considered and the health indicators chosen to measure health expectancy. Evidence of gender-based differentials and a socioeconomic gradient were found in all studies. A remarkable heterogeneity in the choice of health indicators used to compute health expectancy emerged as well as a non-uniform way of defining same health conditions.

**Conclusions:**

Health expectancy is a useful and convenient measure to monitor and assess the quality of ageing and compare different groups and populations. This review showed a general agreement of results obtained in different studies with regard to the existence of inequalities associated with several factors, such as gender, education, behaviors, and race. However, the lack of a standardized definition of health expectancy limits its comparability across studies. The need of conceiving health expectancy as a comparable and repeatable measure was highlighted as fundamental to make it an informative instrument for policy makers.

## Introduction

Between 2000 and 2050 the proportion of the world's population over 60 years is expected to double from about 11 percent to 22 percent, according to WHO estimates [1]. Global ageing has a major influence on disability trends. Higher disability rates among older people reflect an accumulation of health risks across a lifespan of disease, injury, and chronic illness [2]. The rapid increase in population ageing and the longer survival to chronic conditions has raised the question of how healthily the gained years of life will be spent. Life expectancy has been used as an indicator of population health for long time, but with the completion of the "Epidemiological transition" and infectious diseases replacing chronic diseases [3], mortality has ceased to be as tied to health as it was before and life expectancy does no longer capture population’s health. This has led towards the development of a new measure, and in the 1960s the concept of health expectancy was first introduced [4,5]. Health expectancy (HE) is a summary measure of a population’s health (SMPH) that expresses the average number of years that a person can expect to live in "full health"[6]. This very basic definition of HE contains both the strength and limitation of this indicator.

The main advantage of health expectancy is that it captures both the “quantity” and “quality” of life dimensions of health. As it combines information on mortality and health, it is more informative than life expectancy to forecast expected healthcare costs and assistance needs, as well as to plan pension systems and set age of retirement in a sensitive way; and from a societal point of view, it helps to anticipate possible changes in social participation and social inclusion at older ages. Information on health expectancy can be analyzed together with information on life expectancy, and their trends related to each other and compared to assess whether the proportion of life spent unhealthily is expanding due to the longer survival of individuals suffering from chronic diseases [7], or compressing due to the postponement in the onset of morbidity [8], or is associated with a redistribution of diseases resulting in a dynamic equilibrium [9]. HE and the other SMPH have been also largely used to compare different populations and identify and quantify health inequalities within and between sub-groups; for example between genders, social classes, behavior-based groups, or across different countries. Comparisons in HE of different populations can be used to evaluate the performance of different health systems and to identify the determinants of inequalities between populations [10].

On the other hand, one of the drawbacks of using this measure descends from the fact that the whole concept of health expectancy depends on the interpretation given to “full health”. This has generated a variety of ways of expressing health expectancy, differing depending on the health indicators used to capture “full health”. Commonly used terms are disability-free life expectancy, active life expectancy, healthy life expectancy and years of healthy life. These measures are not directly comparable and one is not preferable to another. Authors often define health depending on the availability of the information they have access to or on the objective they want to pursue. The problems arising from this lack of standardization have already been pointed out [11] and efforts to find a global and homogeneous definition of health and disability at international level are growing. In 2001 the World Health Organization (WHO) proposed a conceptual framework for describing functioning and disability: the International Classification of Functioning, Disability and Health (ICF), which conceives and organizes disability as a combination of three components: Impairment, Activity and Participation. Descending from ICF, a more parsimonious conceptualization and measurement of disability was proposed by the Washington Group on Disability Statistics, which uses a short set of six questions to assess disability [12]. Following the same direction, an increasing number of health surveys-such as the Health and Retirement Study (HRS), English Longitudinal Study of Ageing (ELSA), Survey on Health Ageing and Retirement in Europe (SHARE)- run in different countries and sharing similar intents have been trying to harmonize themselves and ask the same questions for the most important health items, in order to facilitate the comparability of studies at international level. Among these health indicators the most commonly used are activities of daily living (ADLs), which are basic activities that are necessary to independent living, such as eating, bathing and dressing; instrumental activities of daily living (IADLs) which are activities that involve aspects of cognitive and social functioning, including for example shopping and cooking; and self-rated health (SRH) which is a general self-evaluation of health status and is usually assessed on a scale ranging from excellent to poor. The efforts made to standardize the concept of health, however, have not been transferred yet to build an indicator of health expectancy that is as much homogeneous and universal. The heterogeneity of the measures falling under the wide umbrella term of “health expectancy” will be illustrated while presenting the results of this review and the implications of this lack of agreement will be discussed further after showing the current state of affairs.

The widespread use of SMPH to measure and compare population’s health and the need of shed some light on the complexity and the uncertainty surrounding the concept of health expectancy have motived this systematic literature review. More specifically, the review seeks to collect and give systematic appraisal of studies exploring socioeconomic and demographic inequalities in health expectancy, focusing on the older population. Within this general aim, the first objective was to appraise the extent of such inequalities and observe which factors have been considered and which remain unexplored. The second objective was to assess the strengths and limitations of using HE to study inequalities in healthy survival, especially in terms of comparability of results across different studies and populations.

In the next section our search strategy is described and the processing and selection of references are explained. Then, findings are organized and presented by type of inequality considered, type of health indicator used and methods applied to estimate health expectancy. Finally, gaps in understanding HE are outlined and limitations acknowledged.

## Methods

### Search strategy

Two possible sources were considered for the literature review: 1) electronic databases, and 2) references in selected articles. Electronic databases were the main source: we used Web Of Science, Medline and Embase.

The search was developed to combine terms referring to outcome, exposure and participants of interest (Table 1). The general term *health expectancy* was declined using a number of expressions all referring either to physical or general health, such as disability-free life expectancy (DFLE), active life expectancy (ALE), healthy life expectancy (HLE). As the family of SMPH is quite large and heterogeneous, given the size of the literature that uses this measure, we decided to focus only on physical and self-reported health combined with mortality. We did not include studies focused on mental functions and cognitive impairments, even if these may cause disability at later stages. The target population-the study participants- of this search was older individuals. We did not use any specific age threshold to define old age, but general expressions commonly applied to identify older adults and the most frequently used age groups, such as 65+, were included among the keywords. The exposures of interest were socioeconomic and demographic variables. We used general terms, such as ‘socioeconomic factors’, as well as specific components, e.g. education or income, and a wide range of demographic terms including gender, marital and parental status, race and ethnicity. Table 1 shows text-words and-where available- MeSH terms used in the systematic search.

**Table 1:** Search strategy: keywords and MeSH terms for systematic literature review.

Concept	Keywords^a^	MeSH termsᵇ
1. Outcome: Health Expectancy	“health* life expectanc*” OR “active life expectanc*” OR “disability-free life expectanc*” OR “health* life year*” OR “Health adjusted life expectancy” OR “disability adjusted life expectancy” OR “disability life expectancy” OR “dependent life expectancy” OR “life expectancy with disability*” OR “health* expectancy*	**Medline:** Life expectancy, Mortality, Health Status Indicators, Health Status, Health Status Disparities, Disabled Persons, "Activities of Daily Living", Life Tables, Morbidity. **Embase:** life expectancy, mortality, health, health status, health disparity, disabled person, daily life activity, life tables, morbidity, disability
2. Participant: Older population	"old* age*" OR “old* adult*” OR “old* population” OR “old* people” OR "aged 50" OR "aged 60" OR "aged 65" OR "elderly people" OR “elderly population” OR “elderly” OR “senior*”	**Medline:** Aged, Middle Aged, Adult, Aged, 80 and over, Age Factors, Aging. **Embase:** aged, middle aged, adult, very elderly, aging
3a. Exposure—Socioeconomic subgroups	“social class*” OR “education*” OR “socioeconomic factors” OR “socioeconomic status” OR “socioeconomic position” OR “Occupation*” OR “income” OR “employ*” OR “housing tenure” OR “deprivation area” OR “deprivation index” OR “poverty area*” OR “area* of deprivation”	**Medline:** Socioeconomic Factors, Occupations, Income, Educational Status, Employment, Social Class, Social Conditions. **Embase:** socioeconomics, occupation, income, education, adult education, employment status, social class, social status
3b. Exposure—Demographic subgroups	“sex” OR” gender” OR “marital status” OR “parental status” OR “fertility” OR “number of children” OR “race” OR “ethnicit*”	**Medline:** Sex Factors, Family Characteristics, Family Relations, Child, Ethnic Groups, Population Groups, Male, Female, Gender Identity. **Embase:** sex, parenthood, marriage, cohabitation, ethnic group, ethnicity, male, female, gender

ᵃ used in Web of Science, Medline and Embase.

ᵇ used in Medline and Embase.

* truncation symbol.

Searches combined with AND: 1 AND 2 AND 3a, 1 AND 2 AND 3b.

### Selection strategy

Titles and abstracts of all references identified in the search were screened applying exclusion and inclusion criteria. The references shortlisted from this first screening were read in their entirety and selection criteria re-applied to full texts. The lists of references of the resulting studies were checked to ensure that all relevant articles were included in the search. Inclusion and exclusion criteria were set according to the definitions of the outcomes, exposures and participants and depending on the type of study (Table 2). As for the outcome, studies which did not combine mortality and health in a single indicator were not included, as well as those focused on mental health or cognitive-impairment free life expectancy. Analyses considering only the impact of specific diseases on HE (e.g. diabetes) were not included either. Studies focused on general populations, not exclusively targeted on the elderly, were included only if estimates of HE were provided also for selected old ages. Finally, studies had to consider at least one risk factor (exposure). Most of the studies stratified their sample by gender, and this was considered satisfactory to meet the inclusion criterion. When data were available only at the macro level (e.g. municipality, region) and an ecological approach adopted, references were discharged. All quantitative studies and reviews meeting the criteria were included, while conference abstracts, commentaries and editorials excluded.

**Table 2:** Selection strategy: inclusion and exclusion criteria.

Concept	Inclusion criteria	Exclusion criteria
Outcome: Health Expectancy	Studies estimating any of the outcomes listed in Table 1	Excluded: studies not centred on the outcome of interest (i.e. mortality, health status, cognitive-impairment free life expectancy)
Participants: Older population	Studies focused on older population; studies on general population where estimates of health expectancy were provided also for selected old age groups	Excluded: studies not focused on older adult population
Exposure: a. Socioeconomic subgroups. b. Demographic subgroups	Analysis considering any of the risk factors listed in Table 1	Macro level analysis, ecological studies
Type of study	English, quantitative studies, reviews	Conference abstracts, commentaries, reports and editorials

### Analysis

The findings of the studies included in this review were synthesized in a narrative format, and organized adopting different perspectives. Data were extracted using a customized template based on the PICOS statement and developed in Microsoft Excel including the following items: author, year, setting and country, participants, exposure, outcome, measure of outcome, methods, and source of data (S1 Table). Consequently, we organized findings according to the exposure of interest. Then, we distinguished studies according to the definition of HE they adopted and the measures used to estimate it. Finally, we organized findings based on the methods used to estimate HE. Results were reported following the Preferred Reporting Items for Systematic Reviews and Meta-Analyses (PRISMA) [13] (S2 Table). The review protocol was not registered.

### Methods of estimating Health Expectancy

Two approaches are usually adopted to estimate HE: these are cross-sectional or longitudinal methods. The first typically employs prevalence-based life tables, also known as Sullivan’s Method [5]. The second is based on incidence rather than prevalence and often relies on increment-decrement life table methods, or multistate life tables (MSLT).

Sullivan (1971) provided the first calculation of DFLE. The Sullivan’s method is the most commonly used (see for example Cambois et al.[14]; Crimmins and Saito[15]) because of its simplicity and because it can be applied also to cross-sectional data. Forty-five of the references collected in this systematic search used Sullivan’s method. These are cross-sectional studies and studies comparing HE at different points in time.

The other approach to estimate HE is based on MSLT. MSLT takes into account the possibility of returning to a state of health (or to a state of disability) using incident rates instead of prevalence. In situations where longitudinal data are available, the incidence-based MSLT is preferred because it most accurately reflects the impact of current conditions (i.e., disability onset, recovery, and mortality) on the evolution of the target population. The MSLT model is an extension of the simple period life table that underlies standard life expectancy estimates, and it is the preferred method in analysis of health changes over time (Cai [16], p.4). Thirty-six references in our search used MSLT to compute HE.

When prevalence and incidence remain constant between periods Sullivan’s method and multistate life table have been found to produce similar results [17]. When either prevalence or incidence vary, Sullivan’s method may underestimate (or overestimate) health expectancy because it produces estimates based on past (as opposed to current) probabilities of becoming unhealthy.

## Results

**Fig 1:**
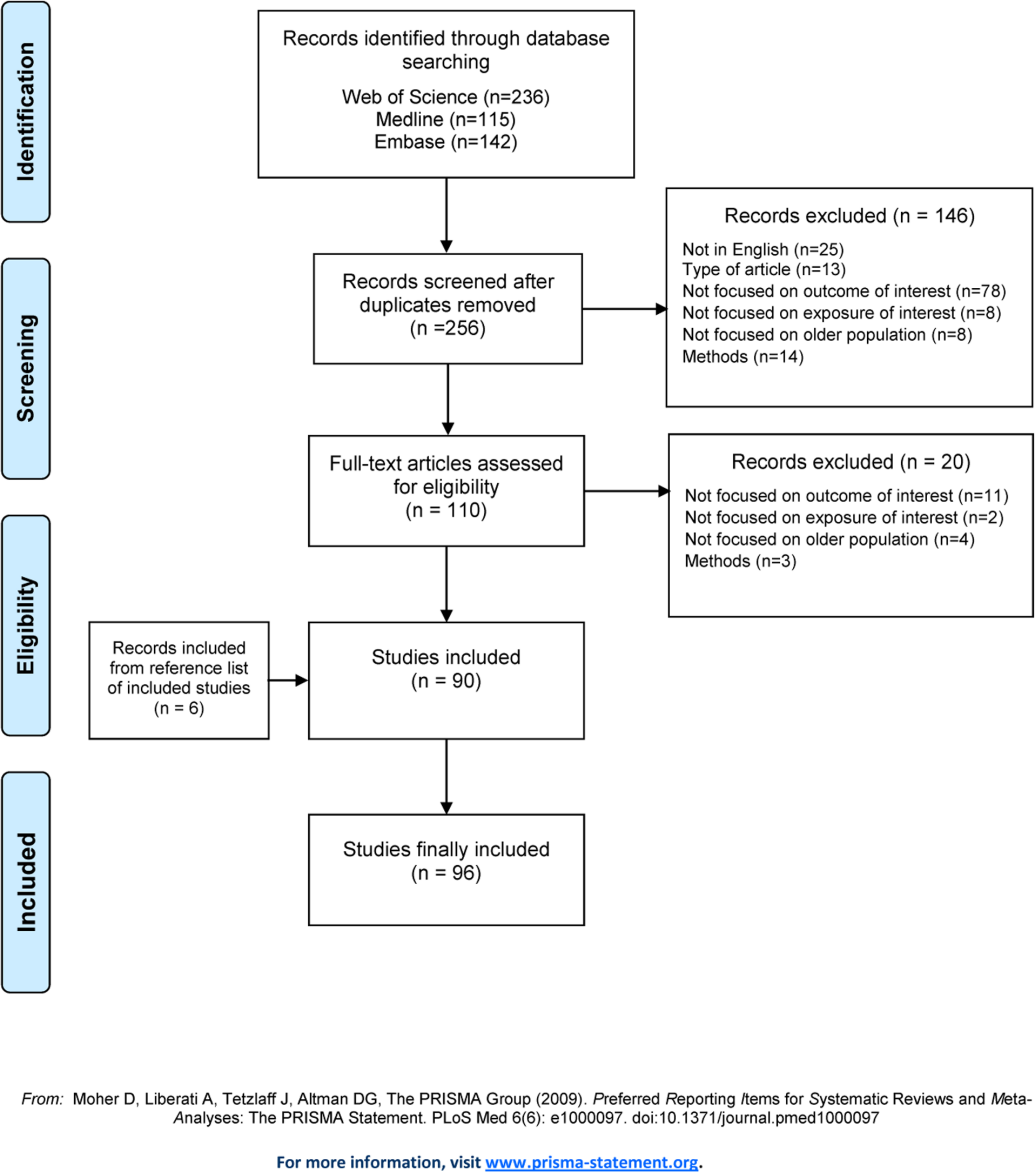
Flow diagram of systematic review.

From the combination of the three databases, 256 references were identified and screened after deduplication (Fig 1). After reviewing titles and abstracts and applying inclusion and exclusion criteria, 110 references were read in full text and 90 selected (a list of the 20 studies considered for inclusion, but finally excluded is provided in S3 Table). Checking the lists of references of these studies, we identified 6 additional articles that were also included. Of the 96 references composing this systematic review, 33 were focused only on gender-based inequalities; the remaining 63 considered gender along with other exposures.

Findings from the 96 selected references were organized according to two leading perspectives: the type of inequalities considered and the health indicators chosen to measure health expectancy.

### Inequalities in Health Expectancy

#### Gender-based inequalities

All studies selected in this literature review, but one, stratified individuals by age and gender (only Crimmins and colleagues [18] did not include gender in their analysis). All results showed evidence of a ‘gender paradox’. The gender paradox in health and mortality was first observed in the mid 1970s [19,20] and consists in the finding that women live longer than men, but tend to have worse health than males. Gender difference in health expectancy can be assessed as the difference between males and females in the number of healthy years or in the proportion of healthy years on total life expectancy. All studies found that females spend a larger proportion of their life in disability than men, but when it came to compare women and men in terms of the healthy-years gap, findings differed across studies. In most cases women were found to live more years in good health than men at every age, but to have a smaller proportion of health expectancy due to their longer survival. For example, in the United States, in 1997 TLE at age 65 was 18.6 years for women and 12.6 years for men, and ALE was 16 and 11.2 years respectively, meaning that women could expect to live 86 percent of their life active and men 88.9 percent [21]. On the contrary, few studies [22,23] found that women’s HE was shorter than men’s HE not only as a proportion of TLE, but also expressed as number of expected healthy years. For example, Sauvaget et al. [24] found that TLE at age 75 in the UK in 1993 was 10.6 years for women and 9.1 years for men, and ALE was 3.1 years for women (corresponding to 29.2 percent of TLE) and 4.6 years for men (corresponding to 50.5 percent).

Whether gender inequalities remain constant as age increases or shrinks at older ages is not clear. According to Minicuci and Noale [25] the ‘gender paradox’ applies to each age group and for any severity of disability; Konno and colleagues [26] found that TLE is longer among women than men until the age of 70 years, but gender-gap in life expectancy declines at older ages, while the gender-gap in the proportion of ALE on TLE increases at older ages, with men enjoying at every age over 90% of their life spent being active and women’s proportion of active life falling from 90% at the age of 65 to 79% at the age of 85. On the contrary, other studies [27–29] found evidence of shrinking gender gap in TLE and HLE at older ages.

Many arguments have been proposed to explain the ‘gender paradox’ in mortality and disability. In general, gender differences are largely due to mortality differences favoring women, rather than differences in the onset of disability [30]. Higher disability prevalence among women may be a function of longer survivorship in disability rather than higher incidence of disability [31,32], in that women have higher prevalence of nonfatal but disabling diseases and men have higher prevalence of fatal diseases and chronic diseases strongly related to mortality [22]. Chang et al. [31] ascribed Japanese women disadvantage in disabilities to gender inequalities in socioeconomic status and disease profiles. The same explanation has been recently adopted for gender differences in health and functional status in Latin America [27]. Robine and Cambois [33] attributed gender differences in ALE at age 65 to gender difference in ALE at earlier ages, and found that the percentage differences were much higher at age 65 than at earlier ages. Muangpaisan et al.[34] found evidence of gender paradox, but when HLE was assessed through SRH, women and men had similar proportions of life expectancy reporting ‘‘being in good health”. This could suggest that women might accept some limitations of health status better than men. Results produced by Ishizaki et al.[28]showed women having longer TLE as well as longer years of dependency in activities of daily living (ADLs) as well as instrumental activities of daily living (IADLs) than men of the same age, but the proportions of physically active life expectancy (PALE) to TLE at any age did not greatly differ between men and women. Jitapunkul et al.[35] found evidence of gender difference in long-term DFLE, but very similar proportions of life expectancy spent unable to self-care between men and women. Tsuji et al.[36] found a marked gender difference in the process of developing and progressing disability, where men experienced disability at a younger ages and at a faster rate than women; the authors argued that this gender-based difference could be attributable in part to the differences between women and men in prevalence and incidence of diseases, where women were found for example to have significantly higher prevalence of arthritis and osteoporosis than men, while stroke incidence was significantly higher in men, as well as heart disease. All these findings would suggest that the strength and validity of gender differentials in HE may vary and depend on the health indicators used to estimate HE and may be explained by a variety of mechanisms, such as inequalities in socioeconomic position and social support and differences in health behaviors and disease profile, all of which would lead to different prevalence of fatal and nonfatal diseases in women and men.

Overall, the gender paradox is widely acknowledged and validated across different studies set in different countries, both high income and low and middle income countries (LMIC). For example, some of the results from our literature review showed evidence of the gender paradox in Brazil[27,29,37–39], Mexico[40], Hong Kong[41], Japan[31,36,42,43], Singapore[44], China[45–50], Bangladesh[23], Thailand[32,34,51–53], United States[22,30,54], Denmark[55–57], England[58,59], Italy[25,60], Bulgaria[61], Turkey [62], France[14,63,64] and across Europe [33,65–68].

#### Race-based inequalities

The studies of racial inequalities in HE included in our search were all set in the United States [15,30,54,69,70] and are mainly focused on Black and White differentials. The common finding is that Whites, as compared to any other ethnicity, enjoy more years in good health, but the gap reduces at older age. Crimmins et al.[30] distinguished Black and non-Black population and found generally non-significant race differences in TLE in the older populations; however Blacks have lower ALE than non-Blacks because of race differences in disability onset and recovery, deriving from socioeconomic inequalities. There is a debate about the existence of a black-white mortality crossover and at what age it would take place. Some argue that racial crossover is due to age misstatement by survey respondents[71]. Crimmins and colleagues [54] found evidence of mortality crossover by age 85 resulting in higher TLE as well as in expected life free of bed disability. Other studies [69,70] found that after age 75 black men and women have an advantage over Whites in both TLE and ALE.

When socioeconomic position (SEP) is controlled for, racial inequalities are larger among lower socioeconomic groups, with low SEP Blacks being the most disadvantaged group [15]. According to Land et al.[70], after stratifying by education, Whites’ advantage in the corresponding unstratified comparisons tends to narrow and Blacks’ disadvantage to decrease. According to Guralnik et al.[69], SEP has a greater association with TLE and ALE than race. Gender inequalities are also consistent across the different race groups: as their white counterpart, black women live longer than black men and spend a higher proportion of their life in disability [30,69].

#### Socioeconomic position-based inequalities

SEP can be measured via different indicators or their combination. The studies included in this literature review identify SEP using alternatively education, income, occupational or social class, area level deprivation, geographical area of residence (urban and rural), housing tenure, social support, or their combination [31,47,72,73]. Whatever the indicator was, all findings agreed that individuals belonging to lower SEP have shorter TLE and enjoy fewer years in good/active health. On the other hand, the strength of this association may depend on the indicator used to measure SEP. There is no agreement on which measure determines the largest SEP-based gap in HE and TLE. Some results suggest that housing tenure and education-based inequalities have the strongest impact, while the inequalities due to income were found not to be significant [72,73]. Other research found income having some strong influences and occupation little impact [31].

The strength of the association between SEP and HE varied across countries. It was substantial in the United States[15], while results pertaining to other countries, such as Indonesia [74], revealed a weaker association. In general, in LMIC high SEP is associated with an expansion of TLE that is accompanied by an expansion of years lived with disability.

It was commonly found that socioeconomic differentials in TLE and HE were larger for men than for women. One of the possible explanations is that women’s SEP may be more a function of their household’s SEP than their individual characteristics [72,73]. Accordingly, Matthews et al. [73] measured women’s SEP through their husband’s job. Chan et al.[31] also claimed socioeconomic inequalities between genders as one of the causes for gender-based inequalities in disability life expectancy (DLE).

SEP-based differences in HE has been generally found to exceed SEP-based differences in TLE, suggesting that inequalities are most pervasive with respect to quality rather than quantity of remaining life [15,64,72,73]. Another common finding is that of a widening of the SEP gap at increasing age [31,72].

#### Education-based inequalities

Since most studies measured SEP through education, special attention was dedicated to HE gap deriving from education-based inequalities. The main advantage of measuring SEP by education is that in most cases individuals have completed their educational path by their early adulthood and this indicator is therefore stable across ages. The vast majority of the studies included in this review confirmed the advantage of being highly educated in terms of both TLE and HLE [15,38,72,73,75]. The consistency of these associations seemed partly to be dependent on the country where the studies were set. Using data from São Paulo and urban areas in Mexico, Beltran-Sanchez and Andrade[76] found some indication of the role of education in influencing HE and TLE in Mexico and Brazil, but no significant educational differences in transition probability (incidence and recovery from disability, and mortality) (p. 827). The analysis of Hidajat et al.[74], showed that education among older Indonesians was associated with increases in life expectancy that was accompanied by longer life with functional problems. On the other hand, studies set in the US [69] found that education impacted both TLE and ALE-and to a greater extent than race- although the differences between higher and lower educated individuals diminished at older ages[77]. In other studies, using multiple indicators for SEP, education was found to be the most strongly predictive measure for HLE and TLE [31,39,72]. Montez and Hayward [78] studied whether educational attainment mediated and moderated the health consequences of early-life conditions. They found that early-life experiences were associated with TLE and ALE, even after adjusting for education. On the other hand, more years of education predicted more years of life and active life, regardless of childhood context (p. 431).

#### Behavior-based inequalities

It is generally recognized that lifestyle factors are associated with both morbidity and mortality. These factors, in particular smoking, alcohol consumption, overweight and obesity, and physical activity, have also been found to be associated with SEP in several populations [79,80] and this represents one of the mechanisms through which SEP might influence health and mortality. However, overall, not many studies have explored the consequences of healthy behaviors on HE among the older population. Results have shown that smoking has a stronger association with mortality than morbidity, while overweight and obesity, and to a lesser extent alcohol consumption, mostly with disability [81]. This difference can be explained by the fact that a high body mass index (BMI), a measure of obesity, is more likely to be associated with non-lethal disabling diseases, whereas smoking is more strongly related to a number of fatal diseases, such as cancer. Weak association of overweight and obesity with mortality but significant association with morbidity was also found in several studies [82–85]; whilst another work [86] showed that mortality rates of overweight and obese participants were not only similar but sometimes better than those of normal weight ones. Flegal and colleagues [87] undertook a meta-analysis of 8 large studies to understand the reasons behind this BMI and mortality paradox. The choice of the reference category for computing the effect measures and the cut points used to define the categories were found to influence estimates and statistical power.

The negative effect of smoking on ALE was confirmed in all studies [77,88,89]. Also differences between heavy and light current smokers and recent and long-term quitters were found in terms of both TLE and ALE. [89]. Only one study in this review considered the association between physical activity and HE [88]. The authors combined this factor with smoking habits and found that the negative effect of inactivity on survival and length of disabled life was comparable or even higher than the effect of smoking.

### Health indicators used to measure Health Expectancy

One of the difficulties in comparing studies on HE derives from the heterogeneity of its definition. HE is a fairly generic measure that can refer to physical as well as cognitive status. This systematic review was focused only on the former, expressed both in terms of general health and disability. Disability however can be defined using a variety of indicators, and in some studies more than one indicator was applied. In the following paragraph and tables we present studies according to the measures adopted to define HE.

Table 3 shows the different health indicators applied to measure HE and the corresponding definition of HE given by the studies included in this literature review. Most of selected references measured HE using ADLs. Forty-two studies adopted exclusively this indicator and in most cases HE was named either DFLE or ALE; other studies used the term ‘Life Expectancy without ADL restrictions’, or ‘Years of life without functional disabilities’, or ‘Life Expectancy without functional problems’. Ishizaki et al.[28] used the expression ‘Physically Active Life Expectancy’ when HE was measured by ADLs, and ‘Instrumentally Active Life Expectancy’ when IADLs were applied. In other cases (seven references) ADLs and IADLs were combined together and HE was named either DFLE or ALE. Fourteen studies used SRH to measure HE and name this latter either ‘Healthy Life expectancy’, ‘Years of Healthy Life’ (YHL) or ‘Life Expectancy in good health’; Lievre et al. (2007) [66] used the term ‘Healthy Working Life Expectancy’ (HWLE), because their work was targeted on working population aged between 50 and 70. Other researchers applied various indicators of functioning and mobility, such as the Barthel ADL index which describes ADLs and mobility using 10 variables[90], and the Chula ADL index [91], composed of five items and conceived to be used in low-middle income countries; other studies referred to the mobility indicators selected by Nagi (1967) [92]that express sensory-motor functioning of the organism, and are indicated by limitations in activities such as walking, climbing, bending, reaching, hearing, etc.; other authors measured HE by sensory function limitations, such as hearing, seeing, walking, etc. The Global Activity Limitation Index (GALI)—which measures long-standing severe disability through a single question such as “For at least the past six months, to what extent have you been limited because of a health problem in activities people usually do?”- was used in five studies. Eight studies measured HE in terms of chronic morbidity, consisting in various chronic or long-term diseases or condition. Finally some authors computed multiple types of HE using different health measures [41,45,53,57,61,63–65,93], this is why the total number of studies included in Table 3 (see column 2) is higher than the total number of studies included in the review. Table 4 presents estimates of HE computed in a sample of four of these studies, to show the variability of HE depending on the health measures adopted as indicator. In all these works TLE and HE were measured at age 65, almost in the same years (2000 and 2002). In all studies one of the measures used to estimate HE was SRH, the lowest estimate was 5.8 years for women in Thailand and the highest 10.3 years for women in Denmark. When health was measured using various questions for ALDs, the proportion of HE on TLE was the highest, for example for men estimates ranged from 84 percent in French men to 95 percent in Thailand (in this study HE was named LE without self-care activity limitations), and for women 77 percent in France and 94.5 in Thailand.

**Table 3:** Type of Health Expectancy by the most commonly used health indicators.

Health indicator (n studiesᵃ)	Type of Health Expectancy	Reference
Activity of daily living (ADL) **(42)**	Disability-free Life Expectancy (DFLE)	Al Snih et al. [82]; Andrade et al. [27]; Cambois et al. [63]; Campolina et al.[94]; Chan et al.[31]; Cheung and Yip [41]; Hayward et al. [95]; Karcharnubarn et al. [53]; Klijs et al. [81]; Matthews et al. [73]; Minicuni et al. [96]; Mutafova et al. [61]; Peres et al. [97]; Santos Camargos et al. [38]; Walter et al. [85]
	Active Life Expectancy (ALE)	Branch et al. [98]; Diehr et al. [86]; Ferrucci et al. [88]; Gu et al. [45]; Gurlanik et al. [69]; Izmirlian et al. [77]; Jiawiwatku et al. [32]; Kai [99]; Katz et al. [100]; Laditka and Laditka [101]; Land et al.[70]; Matthews et al. [93]; Reyes-Beaman et al. [40]; Reynolds et al. [102]; Reynolds and McIlvane [83]; Reynolds et al.[84]; Rogers et al. [103]; Sauvaget et al. [104]; Tian et al.[89]; Tsuji et al. [42]; Yi et al. [105]; Yi et al. [50]
	Physically Active Life Expectancy (PALE)	Ishizaki et al. [28]
	LE without ADL restrictions	Cambois et al. [64]; Jagger et al. [65]
	Years of life without functional disabilities	Yong et al. [44]
	LE without functional problems	Brayne et al. [59]
Instrumental activity of daily living (IADL) **(1)**	Instrumentally Active Life Expectancy (IALE)	Ishizaki et al. [28]
ADL + IADL **(7)**	Disability-free Life Expectancy (DFLE)	Minicuci and Noale[25]; Jagger et al.[106]; Crimmins et al. [18]
	Active Life Expectancy (ALE)	Yong and Sayto [107]; Konno et al.[26]; Hayward et al. [108]; Cai and Lubitz [22]
Various combination of items including: ADL, IADL, Barthel and Chula ADL index, mobility indicators (i.e. NAGI, sensory function limitations, etc…) **(24)**	Disability-free Life Expectancy (DFLE)	Crimmins et al. [54]; Jagger et al. [109]; Jitapunkul et al. [35]; Manton et al. [110]; Minicuci and Noale [60]; Muangpaisan et al. [34]; Sagardui-Villamor et al. [111]; Santos Camargos et al. [39]; Sauvaget et al. [24]; Tareque et al. [23]; Cambois et al. [63]; Beltran-Sanchez and Andrade [76]
	Active Life Expectancy (ALE)	Jitapunkul et al. [52]; Kaneda et al.[72]; Manton et al. [112]; Montez and Hayward [78]; Tsuji et al. [36]; Zimmer et al. [47]; Hidajat et al. [74]
	Healthy Life expectancy (HLE)	Szwarcwald et al. [113]
	LE without mobility limitations	Jeune and Bronnum-Hansen [57]; Karcharnubarn et al. [53]
	Functional Independence LE	Liu et al.[114]; Cambois et al. [64]
Self-rated health (SRH) **(14)**	Healthy Life expectancy (HLE)	Gu et al.[45]; Jitapunkul and Chayovan[51]; Karcharnubarn et al. [53]; Mutafova et al. [61]; Ofstedal et al. [115]; Santos Camargos et al.[29]; Yong and Saito [43]
	Years of Healthy Life (YHL)	Diehr et al.[116]; Diehr et al. [86]
	LE in good health	Bronnum-Hansen[55]; Cambois et al. [64]; Jagger et al.[65]; Jeune and Bronnum-Hansen [57]
	Healthy Working Life Expectancy (HWLE)	Lievre et al. [66]
Global activity limitation index (GALI) **(5)**	Disability-free Life Expectancy (DFLE)	Majer et al.[67]; Cambois et al. [14]
	Healthy Life expectancy (HLE)	Crimmins and Saito [15]
	Active Life Expectancy (ALE)	Robine et al. [33]
	LE without GALI	Cambois et al. [64]
Chronic conditions **(8)**	Years of life w/ and w/o diseases	Yong et al.[44]; Cambois et al. [64]
	Disease-free Life Expectancy	Gu et al. [45]
	LE free of impairment	Jagger and Matthews [58]
	Chronic Morbidity-free Life Expectancy (CMFLE)	Jagger et al. [65]; van den Bos et al.[117]; Cheung et al. [41]; Deeg et al.[118]

ᵃ number of studies does not sum up to the total number of studies in the review, as some studies are counted more than once because several measure of HE were estimated.

**Table 4:** Example of studies estimating multiple types of HE at age 65.

		Women		Men	
Study—Setting, Year	HE	yearsᵃ	% of TLEᵃ	yearsᵃ	% of TLEᵃ
Cambois et al.—France, 2002/03	TLE	20.5	100	16.9	100
	w/o ADL restrictions	15.8	77	14.2	84
	w/o GALI	12.2	59	11.2	66
	in good perceived health	8.2	40	8	47
	w/o functional limitations	6.6	32	6.7	40
	w/o chronic diseases	5.4	26	5.4	32
Gu et al. (2009)—China, 2002	TLE	16.85	100	14.05	100
	w/o ADL restrictions^1^	15.03	89.19	13.08	93.11
	in good perceived health	7.74	45.93	7.17	51.06
	w/o chronic diseases	6.27	37.2	5.69	40.54
Jeune et al. (2008)—Denmark, 2000	TLE	18.1	100	15	100
	w/o mobility limitations^2^	11.9	65.6	12.4	82.4
	w/o GALI	9.5	52.3	8.9	59.1
	in good perceived health	10.3	56.6	9.7	64.3
	w/o communication restriction^3^	15.3	84.3	12.9	85.7
Karcharnubarn et al. (2013)—Thailand, 2002	TLE	17.1	100	15.8	100
	w/o self-care activities limitations^4^	16.1	94.5	15.2	95.8
	w/o mobility limitations^5^	5.4	31.5	8.1	51.5
	in good perceived health	5.8	34.1	6.9	43.4

**ᵃ** Different decimal points according to rounding precision reported in the studies.

^1^Katz's ADL index.

^2^walk 400 m without resting, walk up or down a staircase, carry 5 kg.

^3^read ordinary newspaper print, hear normal conversation, speak with minor or major difficulty.

^4^including only feeding dressing and bathing.

^5^Squatting, carrying thing 5kg, walking 1km, climbing stair2–3 flights, taking a bus/ship alone.

## Discussion

This literature review aimed both at providing a systematic appraisal of studies that investigate socioeconomic and demographic inequalities in HE and giving a critical assessment of this measure. To our knowledge, this is the first review on aggregate/macro level measures of health expectancy in the older population to have been conducted systematically. A literature review on active life expectancy was published in 2002 [71] but it was targeted on women and was not systematic. Crimmins and Cambois [119] collected a number of studies focused on socioeconomic inequalities in HE, but their work was aimed at providing a theoretical explanation of HE and promoting the use of this indicator to design and implement policies.

With respect to the first objective of the review, two main results emerged. The first is the heterogeneity of the indicators and methods used to measure health expectancy and the multiplicity of terms chosen to name it. It was generally observed that DFLE and ALE were used interchangeably, while HLE mainly referred to life expectancy in good SRH, although this was not always the case. Even when HE referred to the same dimension of health (e.g. functional limitations) this was measured using very different indicators. Therefore, the different levels of HE reported across studies may partly depend on the way healthy and unhealthy conditions were defined and not just on the risk factors considered for the analysis. Some studies computed multiple types of HE using different health measures [41,45,53,57,61,63–65,93]. This offered the opportunity to check to what extent the estimates of HE varied depending on the health measure adopted as indicator. In all these studies, the largest proportion of TLE lived healthily was observed when health was measured by limitations in ADLs, and the smallest when chronic morbidity were used [41,45,64,65], while intermediate estimates were obtained when using SRH. The other finding is the homogeneity of the results in terms of the types of inequalities found, such as gender, education, behavior-based disparities. It is interesting then to consider these two findings –the heterogeneity of the concept of health expectancy and the homogeneity of the results by type of inequality- together. Regardless of how health expectancy was measured, women were found to have longer life expectancy and spend a larger proportion of their life in poor health or with disability than men, in every study. Similarly, the existence of an educational gradient affecting positively both life expectancy and health expectancy was confirmed by all studies that considered such exposure; as well as belonging to higher socioeconomic classes was found to lead to longer and healthier life. Findings were consistent across studies and results homogeneous in terms of the direction of inequalities; nevertheless, the actual numbers of years of health expectancy that each category may expect to live was not comparable across different studies. For example, it was possible to generally say that women live a shorter proportion of their life without disability than men, but it was not meaningful to report the precise year gap.

The other aim of the review was to give a critical appraisal of HE as summary measure of population health and discuss the convenience of using it to study inequalities in health and mortality. Two considerations are presented with this matter. First, one of the features in the computation of HE is that analyses are driven by and constrained to the availability of mortality rates or life tables for the specific groups. This has led to the study of inequalities among certain groups only. Hence we found that papers concentrated on investigating inequalities in HE by gender and SEP. However, how gender and SEP may influence HE is still controversial. Some hypotheses have been advanced to explain gender inequalities, for example explaining sex differences as a consequence of the differences in the prevalence of fatal and non-fatal diseases among men and women, and partly ascribing the existence of the ‘gender paradox’ to inequalities in socioeconomic characteristics between men and women. On the other hand, the causes of SEP-based disparities are still unclear. One of the possible mechanisms explaining this association are health behaviors; but not many researchers have been able to study their role among older populations and if they did so, have focused only on some risk factors, such as smoking and overweight, whilst other behaviors such as physical activity and diet have remained almost unexplored. The second consideration –also mentioned in the introduction, before the current state of affairs in literature was presented—pertains to the complexity of the concept of health and disability and the difficulty of measuring it. The findings of this review confirmed the limitations in comparing different studies because of the variety of health indicators used to measure HE, the different estimation methods and the multiplicity of definitions for HE. There is not agreement in the way of defining and conceiving HE and there is not a preferred interpretation either. Furthermore, currently there not seems to be efforts put in place to address this problem.

A final aspect that emerged from this systematic search pertains to the way in which institutionalized population has been considered across various studies. Institutionalized individuals represent an important component of older populations. Data used to estimate HE are mainly drawn from surveys that usually do not include these individuals. Some researchers combined different sources of data to cover the whole (total) population[54,93,106,120], others included hypotheses on the health condition of institutionalized population, assuming for example that they were either as healthy as non-institutionalized individuals[33,65] or all impaired[64]; other studies simply did not include this section of the population into their analysis. This heterogeneous approach to institutionalized population is another possible source of bias to take into consideration when comparing results from different studies.

## Conclusion

The empirical evidence for the joint progress of morbidity and mortality are weak and scarce; and results are contradictory [7–9]. The evolution of health in respect to mortality concerns everyone as an individual perspective and involves the society as a whole –both in high and low middle income countries- because the way in which the older population ages determines the needs for health care and social protection, and impacts the availability of resources for younger generations. Health expectancy is a useful and convenient way of monitoring and assessing the quality of ageing. It allows comparing different groups and populations and identifying disadvantaged categories in order to detect specific factors that can help to promote healthy ageing. This review showed a general agreement of results obtained in different studies with regard to the existence of inequalities associated with several factors, such as gender, education, behaviors, race. Some studies considered more than one factor at the time and found a sort of intersectionality of disparities, identifying particularly frail categories. Interventions should be addressed both to protect specific categories at risk and to generally increase the proportion of life that individuals spend in good health, without disability and being independent. This would mean work in the direction of postponing the onset of disability in the general population, as well as providing external help to carry out basic and complex activities (i.e. ADLs and IADLs) after disability has occurred. To make health expectancy an informative instrument to monitor the quality of ageing and learn class from the past and from the comparison with other populations, this indicator has to be comparable and repeatable. We advocate the need of standardizing health expectancy, both as a concept and in its measurement. This direction has already been taken in surveys by asking same questions for measuring certain health items and in research by assessing the validity of indicators across countries and validating different measures with each other [121,122]. What is still missing is to transfer and apply this to a summary indicator. General guidelines should be drawn to clarify the concept of health expectancy and more research carried out to investigate how best it could be computed. The advantage of using health expectancy to capture population health has produced a vast body of literature that has used this indicator to provide evidence of the existence of inequalities. To use these evidence efficiently and provide more in the future in a consistent way and to make health expectancy a global and informative instrument for policy makers, it needs to be conceived and estimated in a standardized and universal way.

## Supporting Information

S1 Table Descriptive characteristics of included studies. (XLSX)

S2 Table PRISMA guidelines checklist. (DOC)

S3 Table Full population of studies considered for inclusion. (XLSX)
